# A first in man phase I trial of the oral immunomodulator, indoximod, combined with docetaxel in patients with metastatic solid tumors

**DOI:** 10.18632/oncotarget.2357

**Published:** 2014-11-01

**Authors:** Hatem H. Soliman, Erica Jackson, Tony Neuger, E. Claire Dees, R. Donald Harvey, Hyo Han, Roohi Ismail-Khan, Susan Minton, Nicholas N. Vahanian, Charles Link, Daniel M. Sullivan, Scott Antonia

**Affiliations:** ^1^ H. Lee Moffitt Cancer Center and Research Institute, Tampa, FL; ^2^ University of South Florida, Tampa, FL; ^3^ University of North Carolina/Lineberger Cancer Center, Chapel Hill, NC; ^4^ Winship Cancer Institute of Emory University, Atlanta, GA; ^5^ NewLink Genetics Inc, Ames, IA

**Keywords:** Indoximod, 1-methyl-D-tryptophan, immunomodulator, docetaxel, indoleamine 2, 3 dioxygenase

## Abstract

**Background:**

Indoleamine 2,3-dioxygenase (IDO) is an enzyme that tumors use to create a state of immunosuppression. Indoximod is an IDO pathway inhibitor. Preclinical studies demonstrated that indoximod combined with chemotherapy was synergistic in a mouse model of breast cancer. A phase I 3+3 trial was designed to study the combination of docetaxel and indoximod.

**Methods:**

Docetaxel was administered at 60 mg/m^2^ intravenously every 3 weeks dose levels 1-4 and 75 mg/m^2^ for dose level 5. Indoximod was given at 300, 600, 1000, 2000, and 1200 mg PO twice daily continuously for levels 1-5, respectively. Serum drug levels were measured.

**Results:**

Twenty-seven patients were treated, with 22 evaluable for response. DLTs included grade 3 dehydration (level 1), hypotension(level 4), mucositis (level 4) and grade 5 enterocolitis (level 2). Dose level 5 is the recommended phase II dose. The most frequent adverse events were fatigue (58.6%), anemia (51.7%), hyperglycemia (48.3%), infection (44.8%), and nausea (41.4%). There were 4 partial responses (2 breast, 1 NSCLC, 1 thymic tumor). No drug-drug interactions were noted.

**Conclusions:**

Docetaxel plus indoximod was well tolerated with no increase in expected toxicities or pharmacokinetic interactions. It was active in a pretreated population of patients with metastatic solid tumors.

## INTRODUCTION

Indoleamine 2,3-dioxgenase (IDO) is a tryptophan-catabolizing enzyme that inhibits the immune response. IDO was first discovered as an enzyme of the placenta, essential for the prevention of fetal rejection by maternal T cells [1]. It was later determined that IDO played a critical role in the modulation of autoimmunity and transplantation [2]. Because T cells are sensitive to tryptophan deficiency, depletion causes growth arrest in the G1 phase of the cell cycle [3, 4]. IDO can deplete the local tryptophan supply, thereby blocking the proliferation of reactive T lymphocytes [3, 4].

Immune cells are often present at the tumor site, not only to recognize and eliminate malignant cells, but conversely to aid the tumor in evasion of immune destruction [5]. Recent literature has determined that IDO can be used by tumor cells to avoid elimination by the host immune response [6]. It was hypothesized that an inhibitor of IDO would increase the effectiveness of the T-cell response against tumors leading to growth inhibition. Indoximod (D-1-methyl-tryptophan) was developed as an inhibitor of the IDO pathway. The proposed mechanism of action is reversal of the downstream effects of IDO activation through amino acid sensing and mTOR pathways [7]. Preclinical data support the ability of indoximod to reverse IDO-mediated immune suppression [8]. The immunosuppressive activity of IDO leads to an increase in the number of T-regulatory cells, as measured by their Foxp3+/CD4+/CD25+ phenotype. Indoximod has also been shown to reduce the number of T-regulatory cells [9].

In a study with MMTV-Neu mice, researchers looked at the activity of indoximod with and without paclitaxel [10]. Mice with palpable tumors were treated with control vehicle, indoximod, paclitaxel, or indoximod with paclitaxel. A taxane was chosen based on evidence that it increased effector T cells at the tumor site [11]. The combination produced significantly more tumor regression than either agent alone. This synergistic effect was lost when the experiment was repeated in immunodeficient mice, demonstrating that the benefit was dependent on an anti-tumor immune response. Additional preclinical data suggested that indoximod can synergize with other chemotherapy agents, such as doxorubicin and platinum salts [10]. The single-agent phase I trial of indoximod demonstrated very good oral bioavailability and a mild toxicity profile with no significant myelosuppression, and no maximally tolerated dose was identified up to 2000 mg orally twice daily [12]. Based on the preclinical data and good safety profile of single-agent indoximod, we initiated a phase I trial to investigate the combination therapy of docetaxel plus indoximod in patients with metastatic solid tumors.

## PATIENTS AND METHODS

### Patient eligibility

Eligibility for this study included patients with advanced solid tumors, age greater than 18 years, life expectancy greater than 4 months, Eastern Cooperative Oncology Group (ECOG) performance status 0-2, and adequate organ/marrow function. Patients were excluded if they met any of the following criteria: *1*) chemotherapy/radiotherapy within the past 3 weeks, *2*) untreated brain metastases, *3*) uncontrolled concurrent major illness, *4*) current use or previous allergic reaction to L-tryptophan, *5*) active autoimmune disease or chronic inflammatory condition requiring use of steroids or systemic immunosuppressants, *6*) pregnant, *7*) AIDS/HIV infection, or *8*) history of gastrointestinal disease causing malabsorption/obstruction. In addition, patients that received active immunotherapies such as adjuvant interferon less than 1 year prior to enrollment were excluded. There were no restrictions on number of prior lines of therapy, and could have received prior docetaxel in the adjuvant (but not metastatic) setting. Any patient who received prior experimental immunotherapy consisting of targeted monoclonal antibodies was excluded. However, patients who received prior therapy with approved monoclonal antibodies such as bevacizumab, cetuximab, panitumumab, or trastuzumab were eligible. Patients were accrued through the Southeast Phase 2 Consortium consisting of the following locations: H. Lee Moffitt Cancer Center and Research Institute, Billings Clinic Cancer Center, Massey Cancer Center, Lineberger Comprehensive Cancer Center, and the Winship Cancer Institute of Emory University. Both men and women and members of all races and ethnic groups were eligible for this trial.

### Study design

The protocol was approved by the National Cancer Institute's Cancer Therapeutics Evaluation Program and conducted in accordance with all federal and institutional guidelines. All patients provided written informed consent under an Institutional Review Board-approved protocol prior to initiation of any study procedure (University of South Florida IRB, FWA# FWA00001669).

The study followed a 3+3 escalation design to determine the maximum tolerated dose (MTD). The MTD is defined as the highest dose level in which less than or equal to one out of six patients experiences a dose-limiting toxicity (DLT). DLTs were defined as *1*) non-hematological grade 3 or greater toxicity probably attributable to therapy and not to underlying disease, *2*) absolute neutrophil count of less than 500/mL for longer than 7 days or neutropenic fever requiring hospitalization despite the use of white blood cell colony-stimulating factors and dose reduction of docetaxel to 60 mg/m^2^, *3*) grade 4 thrombocytopenia (<25,000/mL), and *4*) dose delay due to hematological toxicity for more than 14 days. Grade 3 hypophysitis and dermatitis were the only exceptions to the DLT rule.

This phase I study evaluated the safety of docetaxel at 60-75 mg/m^2^ in combination with escalating doses of indoximod (Table 1). Dose level 5 was amended after accrual to dose level 4 was completed due to pharmacokinetic data from the monotherapy trial that revealed maximal absorption was reached at 1200 mg PO twice daily.

**Table 1:** Dose escalations and treatments administered

Level	Docetaxel (mg/m^2^)	Indoximod (mg)	No. of Patients Treated	No. of Cycles Administered	Cycles per Patient (median)	Dose limiting toxicities
1	60	300	7	28	3	Grade 3 dehydration
2	60	600	6	35	3	Grade 5 colitis
3	60	1000	6	39	3	
4	60	2000	2	2	1	Grade 3 hypotension,mucositis
5	75	1200	6	16	2	

Indoximod was supplied by the National Cancer Institute's Pharmacy Branch and NewLink Genetics Inc (50 mg and 200 mg hard gelatin capsules). It was administered orally twice daily for 21-day continuous cycles on an empty stomach. Docetaxel was prepared with a single-dose diluent containing 13% ethanol in water and was then administered intravenously over 1 hour. Standard supportive medications, including dexamethasone and anti-histamines as required, were used with docetaxel.

The primary endpoint of this trial was to determine the MTD of the indoximod/docetaxel combination using Common Terminology Criteria for Adverse Events 4.0. Secondary endpoints included the determination of the pharmacokinetic data and the overall objective response rate per Response Evaluation Criteria in Solid Tumors (RECIST) 1.1 criteria.

### Safety evaluations

Complete blood counts and metabolic panels were obtained at baseline and every 3 weeks. Pituitary function tests (thyroid stimulating hormone, free T4, leutinizing hormone, follicular stimulating hormone, and adrenocortical hormone) were obtained at baseline and every 6 weeks. Patients underwent complete physicals and adverse event evaluation once each cycle and as clinically indicated.

### Response evaluation

Overall response rate was determined via the criteria described by the RECIST 1.1 guidelines. Baseline evaluations were conducted within 14 days prior to the start of therapy. Scans were performed within 4 weeks prior to the start of therapy. Patients were then reevaluated every 6 weeks with diagnostic CT scans. The best overall response achieved during study therapy was recorded for each patient. The response data presented herein underwent independent radiology review. The duration of overall response was measured from the time criteria were met for complete or partial response until the first date that recurrent or progressive disease was documented. In patients exhibiting response or disease stabilization, treatment was continued until *1*) disease progression, *2*) intercurrent illness that prevented further treatment, *3*) unacceptable adverse events despite appropriate supportive care, or *4*) patient withdrawal from trial.

### Pharmacokinetic Methods

Validated liquid chromatography triple quadrupole with tandem mass spectrometry (LC/MS/MS) methods were used to determine levels of indoximod and docetaxel in plasma. The methods were validated per ICH/FDA guidelines. Plasma samples were prepared for chromatographic injections by protein precipitation (PPT) in both instances. However, methods were conducted as separate analysis due to the vast differences in analyte characteristics.

Indoximod calibration and quality control samples were made by adding known amounts of indoximod to blank plasma. Indoximod-d3, stable isotope labeled internal standard, was used in the assay. A Sirocco 96-well PPT plate (Waters Corp, Milford, MA) was utilized similar to the manufacturer's suggestion. Samples were injected into a ThermoAccela/TSQ Quantum LC/MS/MS system (Thermo Scientific, San Jose, CA). Mobile phase consisted of water and methanol, both containing 0.1% acetic acid. Gradient pumping conditions were run, and samples were maintained at 4°C in the autosampler during sequences. Indoximod was separated using a Luna C18 (2 × 50 mm, 3 μm) column (Phenomenex, Inc., Torrance, CA). Electrospray ionization was employed, and multiple reaction monitoring was conducted in positive mode utilizing the following molecular transitions; 219 →160 and 222 →163 for indoximod and indoximod-d3, respectively. Peaks were detected and integration was performed with Thermo LC Quan software.

The calibration and quality control samples for docetaxel were made in similar fashion as previous with blank plasma. Docetaxel–d9, stable isotope labeled internal standard, was utilized for this analysis. Protein precipitation was carried out in an Ostro 96-well PPT plate (Waters Corp) according to the manufacturer's suggestion. The same instrument and mobile phase was used as previously described. Samples were maintained at 8°C in the autosampler during sequences, and gradient pumping conditions were used. Docetaxel was separated using a Zobrax SB C18 (2.1 × 50 mm, 3.5 μm) column (Agilent Technologies, San Jose, CA). Electrospray ionization was utilized, and multiple reaction monitoring was conducted in negative mode tracking the following molecular transitions: 806 →672 and 815 →672 for docetaxel and docetaxel-d9, respectively. Once again, LC Quan was used for detection of peaks and integration.

Calibration curves were generated for each run, and patient sample concentrations were back-calculated from the corresponding regression line using LC Quan. The assays are linear from 5 to 2500 ng/mL for both indoximod and docetaxel. Recovery of indoximod from plasma is on average greater than 95%. Inter- and intra-assay variability was less than 9% with a relative mean error of less than 10% for indoximod. Docetaxel average recovery from plasma was determined to be greater than 78%. Docetaxel assay inter- and intra-assay variability was less than 11% with a relative mean error of less than 6%. Plasma concentration-time data for both drugs was analyzed by non-compartmental pharmacokinetic methods using Phoenix WinNonlin 6.3 (Pharsight Corp., Mountain View, CA). Data in the terminal, log-linear phase were analyzed by linear regression to estimate terminal elimination rate constant and half-life. These additional pharmacokinetic parameters were also determined: AUC_0-48_, AUC_0-inf_, C_max_, T_max_, clearance, and volume of distribution.

## RESULTS

### Patient population

Patient demographics are outlined in Table 2. Patients with a variety of cancers were enrolled; however, most primary sites were categorized as non-small cell lung carcinoma (34%) or breast (28%). Eighty-six percent of patients received treatment in the metastatic setting before entering the study. Of the four untreated patients, one withdrew consent during cycle 1 and was unevaluable for response. One of the breast cancer patients was assigned to dose level 1 after relapsing within six months of adjuvant dose dense doxorubicin/cyclophosphamide/paclitaxel chemotherapy. The second breast patient relapsed on adjuvant endocrine therapy and was assigned to dose level 2. The fourth patient was a stage IIIB unresectable lung cancer patient who initially was treated with cisplatin/pemetrexed/vorinostat plus radiation, then had progression of disease and was assigned to dose level 3. Patients received a median of five prior therapies, mostly consisting of chemotherapy. Almost all patients were ECOG performance status 0 or 1.

**Table 2:** Patient and Treatment Characteristics (N=29)

Demographic	
**Sex, n (%)**	
Men	15 (51)
Women	14 (49)
**Age**	
Mean ± SD	53 +/− 10
Median (range)	53
**Ethnicity, n (%)**	
White	25 (86)
Black	3 (10)
Other	1 (3)
Disease type, n (%)	
NSCLC	10 (34)
Breast (4 ER+, 4 TNBC)	8 (28)
Laryngeal	2 (7)
Esophageal	2 (7)
Ovarian	2 (7)
Uterine	1 (3)
Thymus	1 (3)
Liposarcoma	1 (3)
Rectal	1 (3)
Pancreas	1 (3)
**ECOG Performance Status, n (%)**	
0	11 (37)
1	14 (48)
2	4 (13)
**Previous therapies (median)**	
Total	5
Chemotherapy	3
**Previous therapy, n (%)**	
Total	25 (86)
Chemotherapy	24 (83)
Radiation	9 (31)
Hormonal/endocrine	5 (17)
Trastuzumab	2 (7)

### Patient flow

**Figure 1:**
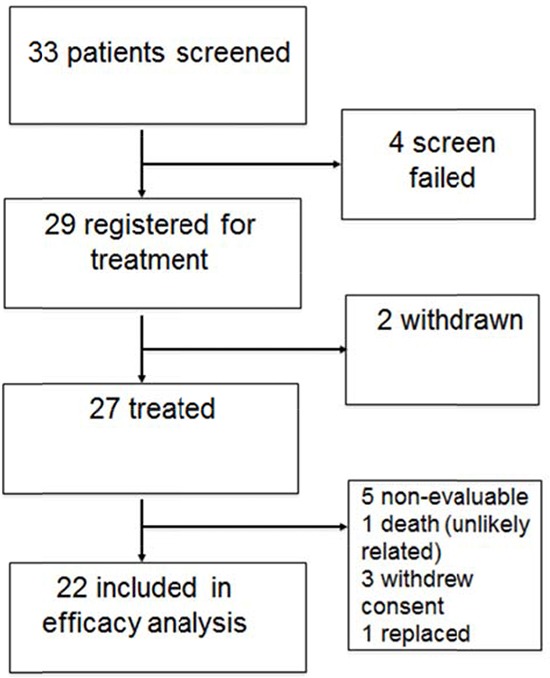
Patient flow diagram

Thirty-three patients were screened for the study with five failing to meet all screening criteria. Twenty-nine patients were then registered for treatment. Two patients withdrew consent during cycle 1, with 27 patients initiating treatment. One patient began treatment, was then found to be ineligible after the first cycle, and was replaced. One patient (dose level 2) expired on cycle 1, day 10 and was not evaluable for response. Three patients withdrew consent during cycle 1 and were not assessed for the response endpoint. Twenty-two patients were included in the response analysis. Figure 1 shows a chart of patient flow through the trial.

### Adverse events

Table 3 lists the frequency of any grade 3, 4, or 5 toxicities that occurred, as well as any grade 1 or 2 toxicity with a frequency greater than 25% regardless of attribution. Adverse events occurring with the highest frequency included grade 1 anemia, fatigue, and hyperglycemia (41%, 45%, and 38%, respectively). Common grade 3/4 adverse effects included neutropenia and febrile neutropenia (both 13%).

**Table 3:** Adverse events by grade regardless of attribution

Toxicity	Grade 1 n(%)	Grade 2 n(%)	Grade 3 n(%)	Grade 4 n(%)	Grade 5 n(%)
**Hematologic**					
Anemia	12(41)	8(28)	1(3)	0	0
Leukopenia	0	0	1(3)	2(7)	0
Lymphopenia	0	0	2(7)	0	0
Neutropenia	0	0	1(3)	3(10)	0
Thrombocytopenia	0	0	1(3)	0	0
**Endocrine**					
Hyperglycemia	11(38)	8(28)	1(3)	0	0
**Infection**					
Febrile Neutropenia	0	0	4(14)	0	0
GI Infection	0	0	1(3)	0	0
Gram Negative Infection	0	0	1(3)	0	0
Pneumonia	0	0	2(7)	0	0
Sepsis	0	0	0	1(3)	0
Skin Infection	0	0	1(3)	0	0
Tooth Abscess	0	0	1(3)	0	0
**Gastrointestinal**					
Abdominal Pain	0	0	1(3)	0	0
Anorexia	8(28)	0	0	0	0
Bowel Perforation	0	0	0	1(3)	0
Colitis	0	0	0	0	1(3)*
Constipation	8(28)	0	0	0	0
Dehydration	0	0	2(7)*	0	0
Diarrhea	8(28)	0	0	0	0
Nausea	10(34)	0	1(3)	0	0
Oral Mucositis	0	0	1(3)*	0	0
Vomiting	8(28)	0	0	0	0
**Metabolic**					
Hypercalcemia	0	0	1(3)	0	0
Hypoalbuminemia	9(31)	0	2(7)	0	0
Hypocalcemia	0	0	1(3)	1(3)	0
Hypokalemia	0	0	1(3)	0	0
Hyponatremia	0	0	4(14)	0	0
**Pulmonary**					
Cough	6(21)	0	0	0	0
Dyspnea	8(28)	0	1(3)	0	0
Pleuritic Chest Pain	0	0	1(3)	0	0
**Renal**					
Increased Creatinine	0	0	1(3)	0	0
Hematuria	0	0	1(3)	0	0
**Vascular**					
Hypotension	0	0	3(10)*	0	0
**Muskuloskeletal**					
Alopecia	8(28)	0	0	0	0
Arthralgia	0	0	1(3)	0	0
Bone Pain	0	0	1(3)	0	0
**Neurologic**					
Peripheral Neuropathy	7(24)	0	1(3)	0	0
Weakness	0	0	1(3)	0	0
**Constitutional Symptoms**					
Fatigue	13(45)	13(45)	0	0	0
**Pain**					
Headache	0	0	2(7)	0	0
Jaw Pain	0	0	1(3)	0	0
**General Disorders**					
Multi-Organ Failure	0	0	0	1(3)	1(3)

### Dose-limiting toxicities

DLTs included grade 3 dehydration at level 1, grade 5 colitis at level 2, and grade 3 hypotension and mucositis at level 4. The grade 5 colitis was caused by sudden mesenteric ischemia leading to sepsis. The death was determined unlikely to be related to indoximod by the site investigators, as well as after thorough safety review by the protocol monitoring committee. Dose level 5 (75 mg/m^2^ of docetaxel + 1200 mg PO twice daily of indoximod) was administered to 6 patients without a DLT and thus was deemed the maximally tolerated dose level.

### Response rate

The overall objective response rates are summarized in Table 4. Four patients achieved a partial response (18%), one patient achieved stable disease for longer than 6 months (4%), nine patients achieved stable disease for less than 6 months (36%), and eight patients had progressive disease (36%).

**Table 4:** Overall response rate in 22 patients included in efficacy analysis

Objective Response Rate	Number of Patients, n (%)
Complete response	0
Partial response	4 (18)
Stable disease > 6 months	1 (4)
Stable disease < 6 months	9 (40)
Progressive disease	8 (36)

**Figure 2:**
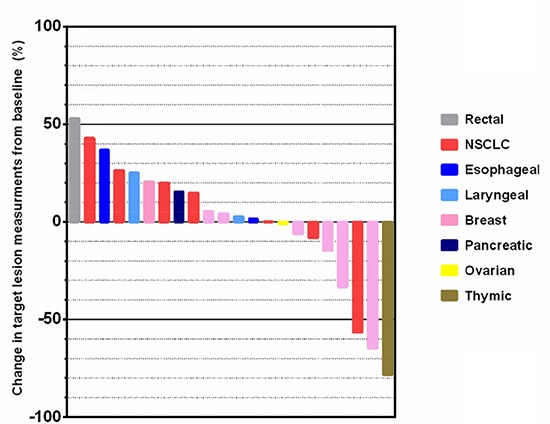
A waterfall plot demonstrating the greatest extent of target lesion measurement changes expressed as a percentage of the baseline measurement.

Figure 2 is a waterfall plot of each patient's best overall response. Four breast cancer patients achieved a reduction in tumor burden (64.8%, 33%, 14.6%, and 6.2% reduction of target lesions). The best of these responders was the untreated breast cancer patient in dose level two who had received only adjuvant endocrine therapy prior to study entry. Two non-small cell lung cancer patients showed a reduction in tumor size (56.5% and 8.2%), as well as one patient with thymic cancer (78.3% reduction). The durations of the objective responses were 15.4 and 5.9 months in the two breast cancer patients. In the lung cancer and thymic cancer patients the duration of response was 7.1 and 5.9 months respectively. It should be noted that the thymic cancer patient discontinued therapy secondary to fatigue and not due to confirmed radiographic progression of his disease. None of the objective responders were previously treated with docetaxel. Five patients showed only a slight increase in tumor burden with less than 10% growth of target lesions.

### Pharmacokinetics

**Figure 3:**
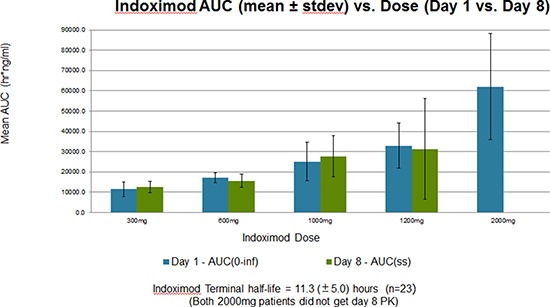
Mean area under the curve (AUC) values of indoximod on cycle 1 days 1 (blue bars) and 8 (green bars) across the five dose levels of indoximod on the x axis Docetaxel did not significantly affect indoximod AUC values at steady state (ss) across all dose levels.

Indoximod pharmacokinetics were similar to results previously reported by our group in the phase I single-agent trial [12]. The half-life was 11.3 (±5.0) hours (n = 23) and AUC was proportional to dose. When comparing day 1 and 8 results, the pharmacokinetics for indoximod were consistent, indicating little to no interaction with docetaxel. When comparing AUC(0-inf) on day 1 with AUC(ss) on day 8, results were nearly the same. Figure 3 represents AUC comparisons. The Cmax of indoximod on day 8 was higher than on day 1 at two dose levels (600 and 1000 mg), but the standard deviation among the patients may have contributed. Dose-independent pharmacokinetic parameter estimates for indoximod can be found in Table 5.

**Table 5:** Indoximod dose-independent pharmacokinetics, day 1 vs. day 8

		Tmax, hours	Vol. of Distribution, L	Clearance, L/hour
**Day 1**	(n=23)	3.6 (±2.0)	634.4 (±463.1)	37.2 (±14.4)
**Day 8**	(n=19)	3.3 (±1.9)	358.7 (±326.5)	43.9 (±42.1)

The pharmacokinetics results for docetaxel do not appear to be affected by indoximod. The results of the two dose levels evaluated were similar to results reported in single-agent studies [13]. Dose was proportional to AUC and Cmax for the study population. Half-life reported here for the entire population, 20.9 (±7.8) hours (n=23), was also similar to previous evaluations. Docetaxel pharmacokinetics by dose level can be found in Table 6.

**Table 6:** Docetaxel pharmacokinetics by dose level on cycle 1, day 1

		t1/2, hours	Cmax, ng/mL	AUC(0-48hr), hr*ng/mL	AUC(0-INF), hr*ng/mL	Vol. of Distribution, L	Clearance, L/hour
**60 mg/m**^2^	(n=20)	19.9 ± 7.4	1660.7 ± 1123.8	3634.9 ± 2487.2	4082.4 ± 2611.8	1145.5 ± 811.4	38.7 ± 26.0
**75 mg/m**^2^	(n=3)	28.1 ± 7.7	1974.6 ± 655.0	3842.6 ± 2299.1	4631.2 ± 2498.5	1805.1 ± 999.6	43.1 ± 20.2

## DISCUSSION

The field of cancer immunotherapy is undergoing a renaissance due to a greater understanding of the complex regulatory pathways that cause tumor-related immunosuppression. Checkpoint inhibitors such as ipilimumab and nivolimumab demonstrated dramatic responses and durable long-term disease control in a fraction of patients with melanoma and non-small cell lung cancer [14-16]. To increase the clinical benefit to patients, combination strategies will need to be explored as a way to overcome immune suppression mediated by other pathways. Indoleamine 2,3 dioxygenase represents one such pathway, and there are efforts underway to see how IDO inhibition can be used to boost the efficacy of other immunotherapies [17]. Another important consideration is that the efficacy of some chemotherapeutics and targeted monoclonal antibodies is in part dependent on their ability to promote an effective anti-tumor response [18-21]. Therefore, combining immunomodulators with chemotherapy or targeted agents to boost this immune response is a logical next step.

This phase I trial provides important information on the feasibility and safety of this chemoimmunotherapy approach using a taxane and an IDO pathway inhibitor. The multiple indications of docetaxel will allow this combination to be tested in a variety of solid tumors that overexpress IDO, such as breast cancer, non-small cell lung cancer, prostate cancer, gastric cancer, and oropharyngeal cancer [8]. Indoximod appears to be ideal from a safety standpoint in that it does not add significantly to the toxicity burden or myelosuppression imposed by the chemotherapy agent. Nadir counts were not routinely obtained due to the high baseline incidence of neutropenia (>90%) from single-agent docetaxel, which would make it difficult to ascertain the impact of indoximod on asymptomatic neutropenia. This is especially the case in a smaller phase I, pretreated patient population with diminished bone marrow reserves. Therefore, our safety evaluations focused on clinically relevant febrile neutropenic events or prolonged myelosuppression that would delay subsequent therapy. The febrile neutropenia rate of 14% was not significantly greater than what has been described in phase III trials, but it should be noted that these historical rates were shown in first-line metastatic patients [22].

The challenge of fully assessing increased toxicity from an investigational compound added to an agent such as docetaxel within a small phase I trial is difficult. However, our ongoing randomized phase II combination trial in first-line metastatic breast cancer patients incorporates interim safety analyses with early stopping rules for excessive toxicity. This will allow a more adequately powered and meaningful assessment regarding whether the combination of docetaxel and indoximod is more toxic than docetaxel monotherapy. As far as the activity of the combination is concerned, any definitive comparative interpretation between the combination and historical activity of docetaxel monotherapy is not possible in this small, heterogeneous group of patients. The fact that 4 of the 8 breast cancer patients showed some degree of anti-tumor activity is an encouraging preliminary signal and warrants further investigation.

A final point that merits discussion is the rationale for the dose selection. While there is a slight increase in the response rate between 75 mg/m^2^ and 100 mg/m^2^ docetaxel in breast cancer patients, there is no significant difference in time to progression between the two doses [22]. Also, the higher toxicity of the 100 mg/m^2^ dose makes it difficult to administer in metastatic patients in combination with other investigational agents [23]. Many combination trials have used the 75 mg/m^2^ docetaxel dose as their preferred recommended phase II/III dose in patients with metastatic breast cancer for these reasons [24, 25]. A single 1200 mg dose of indoximod almost totally saturates the gut, and higher doses do not significantly increase peak serum levels. This serves as our justification for moving forward with the recommended phase II dose of 75 mg/m^2^ of docetaxel in combination with twice daily 1200 mg of indoximod. A limitation of this data from a small phase 1 trial is that one cannot exclude the possibility that indoximod could exert better immunomodulatory effects at slightly higher or lower dose levels of docetaxel.

In summary, we found encouraging activity in a pre-treated population of patients with metastatic disease. According to the pharmacokinetic data, there were no drug-drug interactions between indoximod and docetaxel. The combination was well tolerated with a toxicity profile similar to docetaxel alone. The recommended phase II dose is 75 mg/m^2^ of docetaxel every 3 weeks in combination with 1200 mg indoximod PO twice daily. The response and safety data support the conclusion that this combination is feasible to administer and should be investigated further in a larger randomized phase II trial. A randomized, double blind, placebo-controlled phase II study of docetaxel plus indoximod in metastatic breast cancer patients (NLG201) is now accruing patients.
